# Successful Therapeutic Hypothermia in a Patient with Drug-Induced J Waves and Cardiac Arrest: A Case Report

**DOI:** 10.1089/ther.2023.0041

**Published:** 2023-11-30

**Authors:** Jun Sato, Tsukasa Yagi, Erika Shimada, Masashi Kobori, Kazuhiro Watanabe, Tsukasa Kuwana, Nobutaka Chiba, Takeshi Saito, Kosaku Kinoshita

**Affiliations:** ^1^ Division of Emergency and Critical Care Medicine, Department of Acute Medicine, Nihon University School of Medicine, Tokyo, Japan.; ^2^ Division of Cardiology, Department of Internal Medicine, Nihon University Hospital, Tokyo, Japan.

**Keywords:** J wave, therapeutic hypothermia, extracorporeal cardiopulmonary resuscitation, beta-blocker, calcium channel blocker

## Abstract

A 50-year-old man was admitted to our hospital with hypotension and bradycardia after receiving high doses of atenolol, amlodipine, and etizolam. He had a drug-induced J wave on electrocardiography and subsequently underwent cardiac arrest. The patient was successfully rescued by venoarterial extracorporeal membrane oxygenation (VA-ECMO) and a good neurological outcome was achieved with therapeutic hypothermia (TH). In patients with J waves, TH is thought to increase the J waves and cause fatal arrhythmias, but in this case, rapid cooling with VA-ECMO allowed the patient to successfully complete TH.

## Introduction

The European Resuscitation Council and the European Society of Intensive Care Medicine guidelines on temperature control after cardiac arrest in adults recommend active fever prevention for at least 72 hours in patients who remain comatose after cardiac arrest. The guidelines also state that there is insufficient evidence to recommend or oppose temperature control at 32–36°C or early cooling after cardiac arrest (Sandroni et al., 2022). In cases of cardiac arrest in patients with J waves, not only is there insufficient evidence for temperature management at 32–36°C, but it has also been pointed out that hypothermia may exacerbate J waves and cause fatal arrhythmias (Kashiura et al., 2022).

We report a case of drug-induced J waves leading to cardiac arrest after high doses of atenolol and amlodipine, with a good neurological outcome achieved by extracorporeal cardiopulmonary resuscitation and therapeutic hypothermia (TH). To the best of our knowledge, there have been no such reports previously.

## Case History

**FIG. 1.**
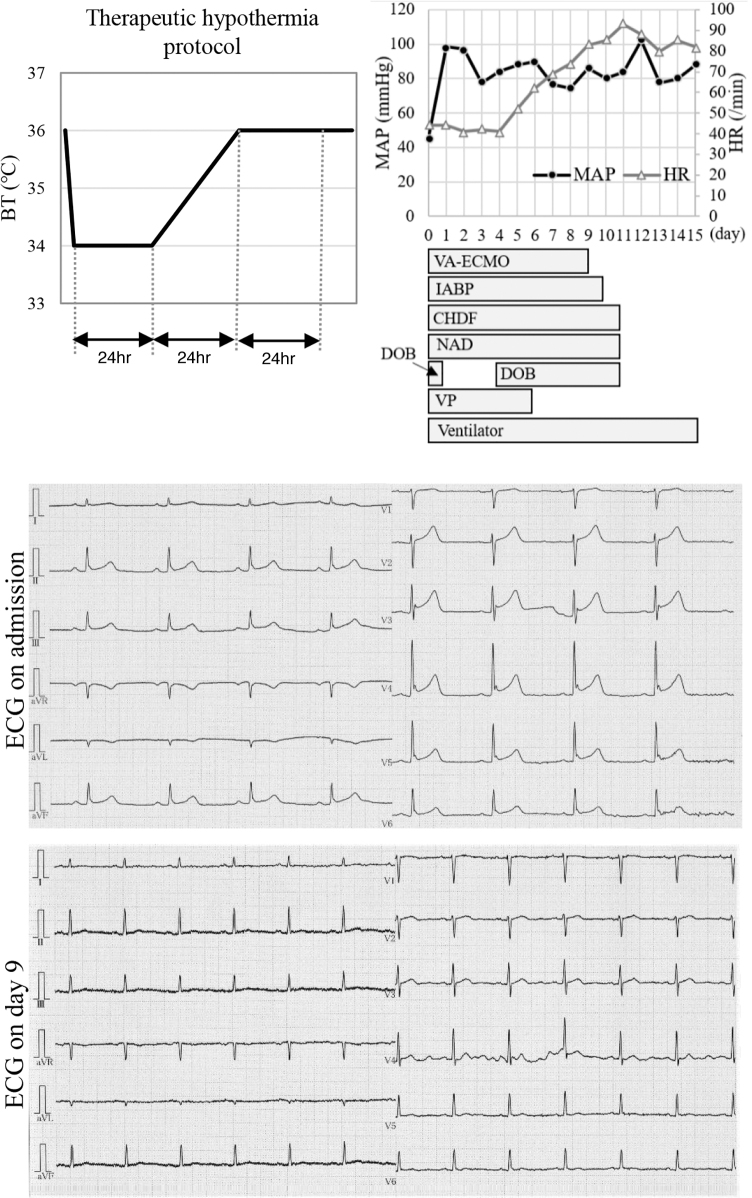
Clinical course of the patient and electrocardiographic trace after admission. BT, body temperature; CHDF, continuous hemodiafiltration; DOB, dobutamine; ECG, electrocardiogram; HR, heart rate; IABP, intra-aortic balloon pumping; MAP, mean arterial pressure; NAD, noradrenaline; VA-ECMO, venoarterial extracorporeal membrane oxygenation; VP, vasopressin.

A 50-year-old man was found after collapsing in his home and transported to our hospital. Hundreds of empty packages of atenolol, amlodipine, and etizolam were found in his home. Physical examination revealed the following: blood pressure, 53/42 mmHg; heart rate, 51/minute; respiratory rate, 23/minute; saturation of peripheral oxygen, 83% on room air; body temperature, 35.9°C; and Glasgow coma scale, 14 points (E3V5M6). Transthoracic echocardiography revealed a left ventricular ejection fraction of 30%. Electrocardiography showed sinus bradycardia with no ST segment and T wave changes, and J waves at II, III, aVF, and V3-6 (Fig. 1). Computed tomography showed pneumonia dorsally in both lung fields.

The patient was treated with levofloxacin for pneumonia and noradrenaline, vasopressin, and dobutamine for septic and cardiogenic shock. We administered atropine for bradycardia; however, the patient's heart rate remained unchanged. His blood pressure did not increase and he experienced pulseless electrical activity. After cardiac arrest, the pupils were 7 mm bilaterally and the contralateral light reflex had disappeared. The lactate level was 5.2 mmol/L at admission, but had increased to 8.5 mmol/L just before cardiac arrest. Cardiopulmonary resuscitation with chest compressions and adrenaline administration was performed, but the patient did not achieve spontaneous circulation and we initiated venoarterial extracorporeal membrane oxygenation (VA-ECMO), intra-aortic balloon pumping (IABP), and TH. The patient was quickly cooled by VA-ECMO to 34°C, maintained at 34°C for the first 24 hours, then warmed to 36°C over 24 hours and held for 24 hours, as per TH protocol (Fig. 1).

It took 27 minutes from cardiac arrest to VA-ECMO implementation. Coronary angiography revealed no significant stenosis in the coronary arteries. Three hours after VA-ECMO initiation, the lactate level increased to 10.2 mmol/L, but 6 hours later, it decreased to 5.8 mmol/L. Fourteen hours after VA-ECMO initiation, the pupils improved to 4 mm bilaterally and the light reflex appeared. The patient also had acute kidney injury and was treated with continuous hemodiafiltration. Figure 1 shows the clinical course. From admission to the fourth day, the patient's heart rate was ∼40 bpm. On the sixth day, the heart rate recovered to 60 bpm, and on the ninth day, the J waves disappeared (Fig. 1). VA-ECMO was weaned on the 9th day and IABP on the 10th day. Vasopressors were discontinued on the 11th day and his level of consciousness improved. The patient was weaned off the ventilator on the 15th day. The patient had a favorable neurological outcome and was transferred to another hospital for rehabilitation on day 36 postadmission.

Informed consent was obtained from the patient for the publication of this case report. This study was approved by the Clinical Research Review Committee of Nihon University School of Medicine (20221107).

## Discussion

In this case, a patient with cardiac arrest with drug-induced J waves was treated with TH using VA-ECMO, resulting in a good neurological outcome. TH has been reported to exacerbate J waves, making our case worth reporting. In cases of cardiac arrest with drug-induced J waves, reaching the target temperature as soon as possible may lead to a good outcome.

In the Targeted Temperature Management-2 trial (Dankiewicz et al., 2021), it took 135 minutes (median) from cardiac arrest to randomization and 3 hours (median) from the start of the intervention to reach 34°C in the hypothermia group. This is considered too long to reach the target body temperature. Hence, significantly more complications of arrhythmias with circulatory collapse in the hypothermia group were observed. In contrast, there are reports of better neurological outcomes with rapid cooling to target body temperature (Arrich et al., 2021). In this case, the target temperature was reached 37 minutes after cardiac arrest; therefore, TH with VA-ECMO may be useful for rapid cooling. The patient still had J waves at 34°C, but no arrhythmias or other complications were observed.

For drug-induced J waves, J wave worsening due to TH may lead to fatal ventricular arrhythmias, and the treatment strategy will depend on whether VA-ECMO is introduced. In cases with VA-ECMO, the patient can complete TH without concern for fatal arrhythmias. In contrast, in cases without VA-ECMO, bradycardia is an exacerbating factor for arrhythmias, so pacing and isoproterenol may be necessary. If the arrhythmia worsens despite these treatments, normothermia may be a better option instead of TH.

In this case, the J wave occurred after high doses of atenolol and amlodipine, and the J wave disappeared in the subsequent clinical course, suggesting that it was drug induced. There are several reports of drug-induced J waves (Noori et al., 2022; Rennyson and Littmann, 2010; Yap et al., 2009). Two hypotheses have been proposed as mechanisms for generating J waves: repolarization and depolarization. The repolarization hypothesis states that in phase 1 of the cardiac action potential, an increase in the transient outward current (Ito) and other outward potassium currents in the epicardial myocardium and a decrease in inward currents cause an increase in repolarization in the epicardial myocardium, resulting in a potential difference between the endocardium and epicardium, and thus in J waves (Benito et al., 2010).

The depolarization hypothesis states that delayed depolarization of the epicardium of the right ventricular outflow tract leads to ventricular late potential, which predisposes patients to arrhythmias (Nademanee et al., 2011). In our case, a calcium channel blocker may have enhanced J waves by blocking inward calcium channels. The bradycardia induced by beta-blockers may also have increased the J waves, as the Ito recovered sufficiently from inactivation to improve the outward current in the epicardial myocardium in phase 1. Correspondingly, Shinohara et al. (2006) reported that J waves are enhanced by propranolol and verapamil administration.

It has been reported that hypothermia slows down the activation of inward calcium channels and increases the relative speed of Ito activation, resulting in an increase in J waves (Aizawa et al., 2019). Therefore, TH may increase J waves in patients with J waves, potentially resulting in lethal arrhythmias. However, this case involved a drug-induced J wave and TH could be successfully completed by maintaining circulation with VA-ECMO until drugs were metabolized.

In conclusion, in this case of cardiac arrest producing drug-induced J waves, TH with VA-ECMO did not result in J wave exacerbation and facilitated a good neurological outcome.

## References

[B1] Aizawa Y, Hosaka Y, Oda H, et al. Dynamicity of hypothermia-induced J waves and the mechanism involved. Heart Rhythm 2019;16(1):74–80; doi: 10.1016/j.hrthm.2018.07.024 30048693

[B2] Arrich J, Herkner H, Müllner D, et al. Targeted temperature management after cardiac arrest. A systematic review and meta-analysis of animal studies. Resuscitation 2021;162:47–55; doi: 10.1016/j.resuscitation.2021.02.002 33582259

[B3] Benito B, Guasch E, Rivard L, et al. Clinical and mechanistic issues in early repolarization of normal variants and lethal arrhythmia syndromes. J Am Coll Cardiol 2010;56(5):1177–1186; doi: 10.1016/j.jacc.2010.05.037 20883924

[B4] Dankiewicz J, Cronberg T, Lilja G, et al. Hypothermia versus normothermia after out-of-hospital cardiac arrest. N Engl J Med 2021;384(24):2283–2294; doi: 10.1056/NEJMoa2100591 34133859

[B5] Kashiura M, Hamabe Y, Moriya T. J-wave syndrome potentially exacerbated by therapeutic hypothermia. Oxf Med Case Rep 2022(3):omac021; doi: 10.1093/omcr/omac021 PMC8931827 35316998

[B6] Nademanee K, Veerakul G, Chandanamattha P, et al. Prevention of ventricular fibrillation episodes in Brugada syndrome by catheter ablation over the anterior right ventricular outflow tract epicardium. Circulation 2011;123(12):1270–1279; doi: 10.1161/CIRCULATIONAHA.110.972612 21403098

[B7] Noori MAM, Fichadiya H, Jesani S, et al. A rare yet morbid complication of cocaine use: Brugada type 1 on electrocardiogram. Cureus 2022;14(4):e24309; doi: 10.7759/cureus.24309 35602832 PMC9122013

[B8] Rennyson SL, Littmann L. Brugada-pattern electrocardiogram in propranolol intoxication. Am J Emerg Med 2010;28(2):256.e257–e258; doi: 10.1016/j.ajem.2009.05.020 20159410

[B9] Sandroni C, Nolan JP, Andersen LW, et al. ERC-ESICM guidelines on temperature control after cardiac arrest in adults. Intensive Care Med 2022;48(3):261–269; doi: 10.1007/s00134-022-06620-5 35089409

[B10] Shinohara T, Takahashi N, Saikawa T, et al. Characterization of J wave in a patient with idiopathic ventricular fibrillation. Heart Rhythm 2006;3(9):1082–1084; doi: 10.1016/j.hrthm.2006.05.016 16945806

[B11] Yap YG, Behr ER, Camm AJ. Drug-induced Brugada syndrome. Europace 2009;11(8):989–994; doi: 10.1093/europace/eup114 19482855

